# A Novel Inhibitor Prevents the Peripheral Neuroparalysis of Botulinum Neurotoxins

**DOI:** 10.1038/srep17513

**Published:** 2015-12-16

**Authors:** Domenico Azarnia Tehran, Giulia Zanetti, Oneda Leka, Florigio Lista, Silvia Fillo, Thomas Binz, Clifford C. Shone, Ornella Rossetto, Cesare Montecucco, Cristina Paradisi, Andrea Mattarei, Marco Pirazzini

**Affiliations:** 1 Department of Biomedical Sciences, Via U. Bassi 58/B, 35121, Padova, Italy; 2 Italian National Research Council Institute of Neuroscience, University of Padova, Via U. Bassi 58/B, 35121, Padova, Italy; 3 Histology and Molecular Biology Section, Army Medical and Veterinary Research Center, Via Santo Stefano Rotondo 4, 00184 Roma, Italy; 4 Institut für Biochemie, OE 4310, Medizinische Hochschule Hannover, 30623 Hannover, Germany; 5 Public Health England, Porton Down, Salisbury, Wiltshire, SP4 OJG, UK; 6 Department of Chemical Sciences, University of Padova, Via F. Marzolo 1, 35131 Padova, Italy

## Abstract

Botulinum neurotoxins (BoNTs) form a large class of potent and deadly neurotoxins. Given their growing number, it is of paramount importance to discover novel inhibitors targeting common steps of their intoxication process. Recently, EGA was shown to inhibit the action of bacterial toxins and viruses exhibiting a pH-dependent translocation step in mammalian cells, by interfering with their entry route. As BoNTs act in the cytosol of nerve terminals, the entry into an appropriate compartment wherefrom they translocate the catalytic moiety is essential for toxicity. Herein we propose an optimized procedure to synthesize EGA and we show that, *in vitro,* it prevents the neurotoxicity of different BoNT serotypes by interfering with their trafficking. Furthermore, in mice, EGA mitigates botulism symptoms induced by BoNT/A and significantly decreases the lethality of BoNT/B and BoNT/D. This opens the possibility of using EGA as a lead compound to develop novel inhibitors of botulinum neurotoxins.

The most potent human poisons are the botulinum neurotoxins (BoNTs), which are neurospecific metalloproteases acting inside peripheral nerve terminals. They are synthesized by different species of Clostridia and have been grouped in seven serotypes (BoNT/A to/G) based on their immunological properties. All known BoNTs act by interrupting the release of neurotransmitter acetylcholine at peripheral cholinergic terminals causing a long lasting paralysis that may lead to death by respiratory failure^1^. Nonetheless, mechanically ventilated patients can fully recover in a time period which strongly depends on the toxin serotypes and on the amount of toxin molecules entered in the nerve terminals^2^. According to their extreme potency, and with the fact that they can be easily produced in large amounts, BoNTs are considered potential bioweapons^3^,^4^. On the other hand, due to their neurospecificity, reversibility and lack of diffusion from the site of injection, BoNT/A has worldwide become one of the safest therapeutics used for the treatment of a growing list of human syndromes, characterized by the hyperactivity of peripheral nerve terminals^5^,^6^. BoNTs consist of two polypeptide chains (L and H), kept together by a single disulphide bond. The overall structure can be subdivided in three 50 kDa domains which accomplish different tasks along the mechanism of neuron intoxication^2^. The L chain is the N-terminal domain endowed with metalloprotease activity. The C-terminal domain (HC) is responsible for the neurospecific binding to the presynaptic membrane of nerve endings, whilst the intermediate domain (HN) is involved in membrane translocation of L. The current view of BoNT mechanism of action envisages a first interaction with polysialogangliosides, which mediate the toxin binding to the plasma membrane. This is followed by lateral movements that make possible the encounter with a protein receptor which is the lumenal part of a synaptic vesicle (SV) protein^2^,^7^,^8^. The protein receptor has been identified as synaptotagmin I and II for BoNT/B,/DC and/G^8^,^9^, and SV2 for BoNT/A,/E and F^8^,^10^; the protein receptor for the remaining serotypes remains to be established. This latter binding is preliminary to the internalization of the toxin-receptors complex inside an acidic intracellular compartment whose nature has been identified as SV only for tetanus neurotoxin and for BoNT/A^11^,^12^,^13^. Little is known on the nature of the endocytic vesicles/compartment used by the other serotypes, but considerable evidence indicate that the acidification of its lumen generally triggers a structural change of L and HN together with membrane lipids which ultimately leads to the translocation of the L chain into the cytosol^14^,^15^,^16^. This process is completed by the reduction of the interchain disulphide bond, on the cytosolic side of the acidic compartment performed by the thioredoxin reductase–thioredoxin system^17^,^18^,^19^,^20^ . The released L metalloprotease specifically cleaves one of the three SNARE proteins thereby preventing the Ca^2+^ induced release of the neurotransmitter contained inside SVs^21^,^22^. Many novel BoNTs have been recently discovered and their sequences are present in databases, but many more have not yet been deposited. All known novel BoNTs are classified as subtypes, and indicated with an Arabic number added to the parental serotype (e.g. A2, A3 etc., when their amino acid sequences differ by more than 2.4% from the parental serotype A1)^2^, or as mosaic BoNTs, and indicated with a double capital letter, e.g. BoNT/DC,/CD,/FA, when they are chimeras of the different serotypes. Due to their different origin, BoNT variants exhibit different antigenicity and are neutralized to a different degree by existing serotype specific antisera^23^,^24^. Accordingly, it is possible that the therapy with humanized monoclonal antibodies raised versus a BoNT subtype may not neutralize variants of the same serotype^25^,^26^. This situation calls for increased efforts in the identification of inhibitors effective in preventing the neuroparalytic action of BoNTs irrespectively of their serotype and subtype which could be used without knowing the particular type of BoNT involved. Recently, Gillespie *et al.* (2013), performing a high-throughput screening, identified 4-bromobenzaldehyde N-(2,6-dimethylphenyl)semicarbazone (abbreviated as EGA) as an inhibitor of pathogens that enter cells via intracellular acid compartments^27^. Since BoNTs toxicity is also strictly dependent on the passage through an acidic environment^2^, we decided to test the activity of EGA on BoNT action in the light of the urgency and importance to find inhibitors capable of interfering with the large and still growing number of BoNTs with undefined immunological properties. Here, we focused our attention on BoNT/A and BoNT/B because most frequently associated with human botulism and used in human therapy^1^,^2^. We also considered BoNT/D, which scarcely affects humans^28^, but it is very frequently involved in animal botulism. Here we show that EGA drastically hinders BoNTs activity on neuronal cultures, without interfering with specific steps of their cellular mechanism of intoxication. More importantly, this compound is also very effective in reducing neurotoxicity *in vivo*. Together, our results suggest that EGA represents a new tool for studying BoNTs trafficking and a good candidate for the development of new inhibitors. Notably, we also report an optimized procedure for the synthesis of EGA, which involves milder reaction conditions and provides much higher overall yield than previously reported^29^.

## Results

### High yield synthesis of 4-bromobenzaldehyde N-(2,6-dimethylphenyl) semicarbazone (EGA)

**Figure 1:**
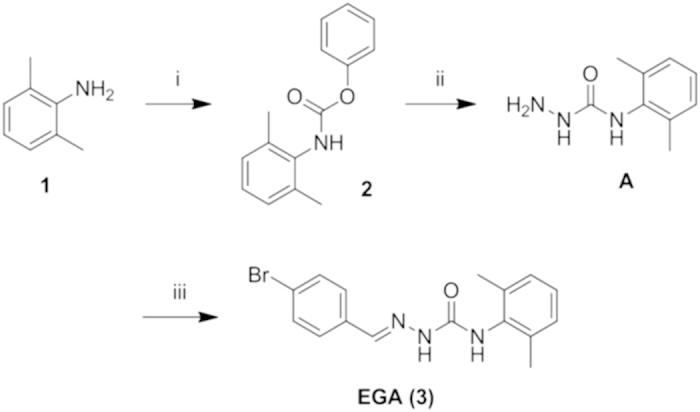
Synthesis of EGA. Synthesis of 2-(4-bromobenzylidene)-N-(2,6-dimethylphenyl)hydrazinecarboxamide (EGA); Reagents and conditions (i) ClCO_2_Ph, Py, DCM (96% isolated yield); (ii) NH_2_NH_2_·H_2_O, DME (iii) 4-bromobenzaldehyde, CHCl_3_ (88% isolated yield, passages ii and iii).

The reported approach by Jung in 2014 for the preparation of EGA has been adapted and improved to obtain higher yields. The synthesis involves the three steps reported in Fig. 1. In the first one (i), 2,6-dimethylaniline (1) is allowed to react with phenyl chloroformate to give the corresponding phenylcarbamate (2), which is next subjected to hydrazinolysis to give semicarbazide (indicated as A) (ii). The final step (iii) is the reaction of A with 4-bromobenzaldehyde to form the desired semicarbazone (3, EGA). The procedure described by Jung *et al.* (2014) involves the isolation of A and rather drastic conditions (acidic solution and high temperature) in the last step, leading to an overall yield of 27%. A much higher overall yield (84%) is obtained by the new procedure that we have devised: 2 is isolated, whereas steps (ii) and (iii) are performed in one-pot without the isolation of A. Furthermore, much milder conditions are used in the last synthetic step. Details are found in the Supplementary information section.

### EGA prevents the botulinum neurotoxins cleavage of SNARE proteins in cultured neurons

**Figure 2:**
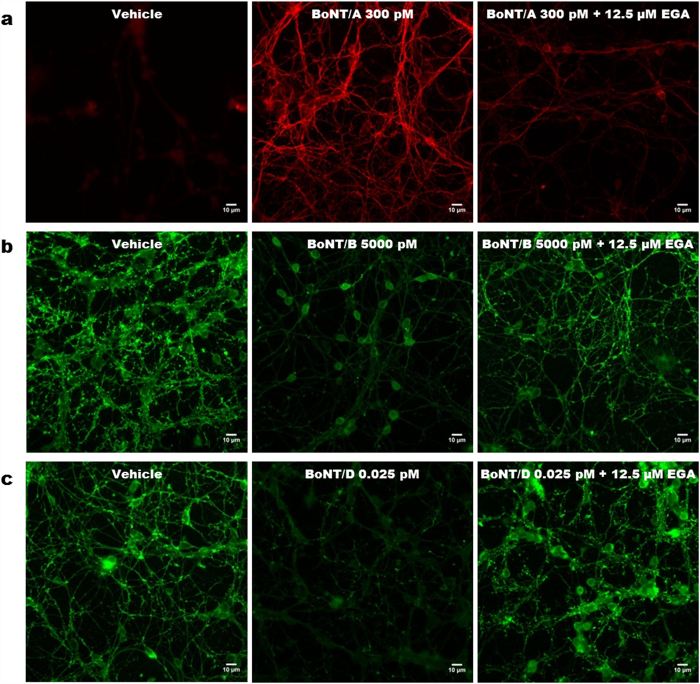
Immunocytochemical evaluation of EGA inhibition against different serotypes of BoNT in CGNs. (**a–c**) CGNs were treated with EGA 12.5 μM or vehicle (DMSO) at 37 °C for 30 min. Thereafter, the indicated amount of BoNT was added for 12 hrs. Samples were fixed and stained with an antibody specific for BoNT/A-cleaved SNAP25 (**a**) or intact VAMP2, (**b,c**). BoNT/A-cleaved SNAP25 was detected with an Alexa Fluor 555 goat anti-rabbit, while VAMP2 with an Alexa Fluor 488 goat anti-mouse. Images shown are representative of three independent experiments. Scale bar, 10 μm.

**Figure 3:**
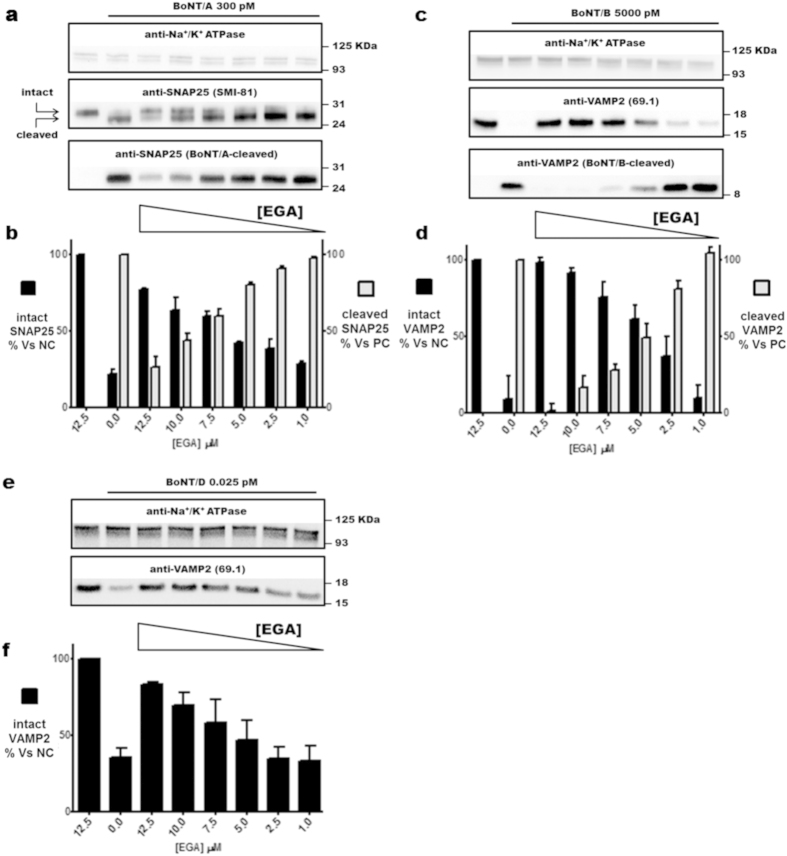
EGA interferes with the BoNT/A,/B and/D toxicity in CGNs. (**a,c,e**) CGNs were preincubated for 30 min with the indicated concentrations of EGA at 37 °C. Where indicated, BoNTs were added at the reported concentrations for 12 hrs. Then cells were lysed and the SNARE content was estimated with the indicated antibodies: (**a**) SMI81 recognizes both the full length and the cleaved form of SNAP25; BoNT/A-cleaved recognizes only BoNT/A-truncated SNAP25; (**c,e**) VAMP2 (69.1) recognizes the intact form of VAMP2 and, BoNT/B-cleaved recognizes only BoNT/B-cleaved VAMP2. In all experiments an antibody against the Na^+^/K^+^ ATPase antibody was used as loading control. Blots are representative of a typical experiment. (**b,d,f**) Densitometry analysis of western blots obtained in (**a,c**) and (**e**) respectively. All data are presented as mean values and error bars indicated the standard deviation obtained from at least three independent experiments.

The use of cultured cerebellar granular neurons (CGNs) offers a simple and rapid way to screen the efficacy of candidate molecules in inhibiting BoNTs activity. The overnight incubation with 0.3 nM BoNT/A induces the cleavage of SNAP25, as assessed by the appearance in immunofluorescence (Fig. 2a, middle panel) and western blot (Fig. 3a, bottom panel) of its truncated form, revealed with a specific antibody. This toxin concentration is sufficient to induce a complete cleavage of its substrate, as evaluated using an antibody recognizing both forms (intact and truncated) of SNAP25 (Fig. 3a, middle panel, SMI-81). Figure 3b shows that such activity is however inhibited by EGA in a concentration dependent manner, with a maximal effect at 12.5 μM. The inhibitory effect, though substantial, is not complete as a small amount of cleaved SNAP25 is still generated (Fig. 2a, right panel and Fig. 3b).

Figures 2b and 3c,d show that similar results are obtained with BoNT/B. Notably, to achieve the substantial cleavage of VAMP2, which is normally concentrated at synaptic contacts (Fig. 2b, left panel), BoNT/B had to be used at a concentration of 5 nM (Fig. 2b, middle panel). Nevertheless, the pre-treatment with 12.5 μM EGA is sufficient to abrogate its cleavage (Fig. 2b, right panel). EGA prevents this toxicity in a concentration dependent manner, as shown by the inhibition of VAMP2 cleavage, determined with two different antibodies (Fig. 3c middle and bottom panels). Interestingly, despite the high amount of toxin used, in this case the effect of EGA, at higher concentration, is complete (Fig. 3d).

The same set of experiments was replicated using BoNT/D. This serotype is the most potent in rodents^28^ and, in CGNs, a minimal concentration (0.025 pM) induces the almost complete cleavage of VAMP2 (Fig. 2c, middle panel). Similar to what found for BoNT/A, we found that EGA substantially prevents the action of this potent neurotoxin (Fig. 2c, right panel), and this inhibition is dependent on the amount of the chemical, as estimated in western blot with an antibody specific for the intact form of VAMP2 (Fig. 3e, bottom panel and Fig. 3f).

Importantly, Figure S1 shows that neurons viability is not significantly affected, even at the highest concentration of EGA used.

### EGA does not interfere with the four basic steps of BoNTs’ mechanism of action

The cellular target of EGA is not known and we investigated whether any of the four main steps of the BoNTs’ cellular mechanism of action is directly impacted by the action of the drug.

**Figure 4:**
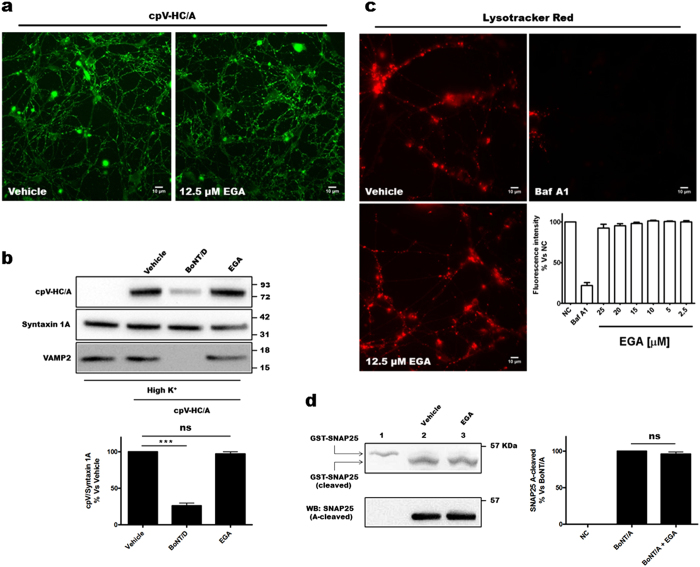
EGA does not inhibit the known steps of BoNT intoxication. (**a**) CGNs were treated with EGA (12.5 μM) or vehicle in culture medium at 37 °C. After 30 min, 100 nM cpV-HC/A was added in high K^+^ buffer for 1 h. Neurons were then washed, fixed, and directly imaged. These images are representative of at least three independent sets of experiments. Scale bar, 10 μm. (**b**) CGNs were treated as in (**a**) with 250 nM cpV-HC/A and then lysed. The cpV-HC/A content was estimated with a GFP specific antibody. Syntaxin 1A serving as internal control was detected with a specific antibody and an anti-VAMP2 was used to assess BoNT/D cleavage. The amount of cpV-HC/A was determined as a ratio to Syntaxin 1A staining taking the value in non-treated cells (vehicle) as 100%. All data are presented as mean values and error bars indicated the standard deviation obtained from three independent experiments (***p < 0.0001; ns – non significant). (**c**) CGNs were treated with vehicle or 12.5 μM EGA or 10 nM Bafilomycin A1 for 30 min at 37 °C. Lysotracker Red was then added and the incubation prolonged for further 90 min. Cells were imaged by fluorescence microscopy. The graph shows the quantification of fluorescence intensity of acid compartments (% versus non-treated neurons) arising from CGNs treated with the indicated amount of EGA. Mean and standard deviation values refer to four different experiments. Scale bar, 10 μm. (**d**) 0.25 μg BoNT/A was reduced in the presence of 12.5 μM EGA for 30 min at 37 °C. 1 μg of GST-SNAP25 was added, the concentration of inhibitor was restored, and the reaction was carried out for 12 hrs at 37 °C. SNAP25 cleavage was assessed by SDS-PAGE and Coomassie staining (top-left panel) or immunoblotting (bottom-left panel) with an antibody specific for the BoNT/A-cleaved form of SNAP25. Lane 1 shows untreated GST-SNAP25. Right panel shows the densitometry analysis of western blots, tacking the value of BoNT/A without EGA (vehicle) as 100%. All data are presented as mean values and error bars indicated the standard deviation obtained from three independent experiments.

The first step of intoxication is the specific binding of BoNTs to peripheral nerve endings followed by their internalization via endocytosis^2^. Given its chemical nature, EGA could in principle intercalate among lipids and alter the properties of the presynaptic membrane, making it less receptive for BoNTs binding. To investigate this possibility, we took advantage of two constructs consisting of the HC domain of BoNT/A and of BoNT/B fused to a fluorescent protein (cpV-HC/A) or tagged by a c-Myc epitope (c-Myc-HC/B), respectively. These chimeras fully maintain the capability of parental BoNTs to bind to the presynaptic membrane of neurons^8^,^30^ and to become endocytosed^12^,^13^. We found that EGA, used at the concentration which displayed the maximum efficacy in protecting CGNs, does not interfere with the binding and the endocytosis of both BoNT/A and BoNT/B, as assessed by the internalization of their respective derivatives which show the same pattern regardless of drug presence (Fig. 4a and Figure S2a). Intriguingly, the two HCs displayed clearly different patterns of staining, suggesting that they may be internalized inside different compartments. Figure 4b and Figure S2b show via western blot analyses the quantitation of the results. We performed this experiment with higher concentrations of HCs to meet the sensitivity requirements of the antibodies used in Western Blot. Consistently, Fig. 4b show that the previous treatment of CGNs with BoNT/D, which cleaves VAMP1/2 thus impairing SVs recycling, significantly decreases the uptake of HC/A, as reported elsewhere^31^,^32^. At variance, the uptake of HC/B was only partially affected by VAMP1/2 cleavage (Figure S2b), leaving open the possibility of a different trafficking of BoNT/B with respect to BoNT/A.

Nevertheless, the fact that BoNT/A and BoNT/B use synaptic vesicle proteins as receptors (SV2A/B/C and Synaptotagmin I-II, respectively) strongly suggests that they exploit SVs for their initial step of endocytosis. Accordingly, we decided to test the possible interference of EGA with SVs dynamics using a well-established assay^33^. As shown in Figure S2c, EGA does not affect SVs endocytosis as an antibody specific for the lumenal domain of the synaptic vesicle marker Synaptotagmin I, is internalized at the same extent as controls. On the contrary, if neurons are previously treated with BoNT/D, the uptake of the same antibody is prevented. The quantitation of the result is shown in Figure S2d. Taken together, these results demonstrate that BoNTs binding and internalization through SV cycling are not perturbed by EGA.

The BoNT exposure to an intracellular acidic compartment is the next essential step for the neuron intoxication by all BoNTs^2^. Using Lysotracker Red DND-99, a highly sensitive probe of acidic organelles in live cells, we found that EGA does not significantly interfere with the maturation of acidic compartments, both within CGNs cell body and along neurites, where BoNTs act (Fig. 4c). At the same time, bafilomycin A1, which prevents BoNTs toxicity by inhibiting the vacuolar-type H^+^-ATPase proton pump^12^,^34^,^35^, completely blocks the acidification of intracellular organelles. This suggests that the essential conditions needed for BoNTs translocation, i.e. an acidic environment, are maintained in the presence of EGA.

The final step of the nerve intoxication, the one responsible for neuroparalysis, is the cleavage of SNARE proteins by the L chain. BoNT/A chops off the last 9 amino acids of SNAP25, whereas BoNT/B and BoNT/D cleave at two different sites VAMP1/2. This proteolytic activity can be easily assayed *in vitro* by using recombinant substrates. As shown in Fig. 4d (left panel), upon reduction of the interchain disulphide bond, BoNT/A cleaves SNAP25, as shown by the shift of its molecular weight in SDS-PAGE (compare lane 1 and 2, upper panels). This activity is however not affected by 12.5 μM EGA (Fig. 4d, compare lane 2 and 3). The same result was obtained using an antibody specific for the cleaved form of SNAP25 and western blotting as a read out (Fig. 4d). Figure S3a and Figure S3b show that similar results were obtained with BoNT/B and BoNT/D, respectively, suggesting that the enzymatic activity of BoNT L chains is not affected by the drug.

### EGA interferes with BoNTs trafficking within neurons

**Figure 5:**
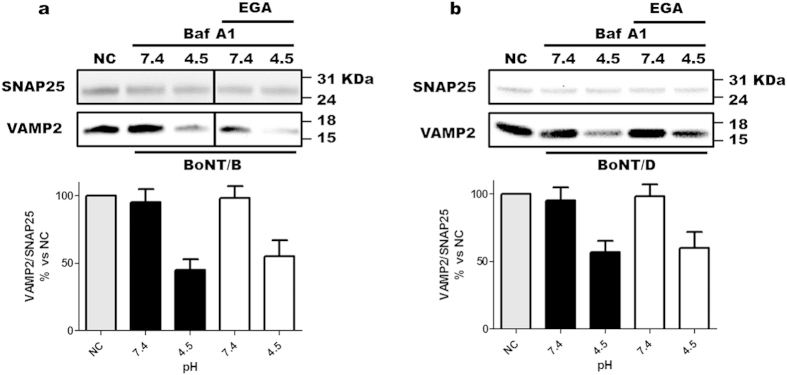
EGA does not inhibit the translocation and the reduction step. (**a**) PC12 cells expressing the lumenal domain of synaptotagmin I on their surface were pre-incubated with a mixture of gangliosides for 24 hrs. Cells were washed and, where indicated, treated with 12.5 μM EGA or vehicle for 30 min at 37 °C. Thereafter, BoNT/B (10 nM) was added in the cold for 15 min. Cells were then washed and incubated with medium A buffered at indicated pH at 37 °C for 10 min, in the presence or absence of EGA. Then, cells were washed and the incubation in culture medium containing 50 nM Bafilomycin A1 and the same concentration of EGA prolonged for 24 hrs at 37° C. The translocation of BoNT/B was assessed by monitoring the cleavage of VAMP2, determined via western blotting (top panel), and quantified (bottom panel) through the densitometry of VAMP2 as a ratio to SNAP25 staining which served as internal control, taking the value in non-treated cells (NC) as 100%. All data are presented as mean values and error bars indicated the standard deviation obtained from three independent experiments (ns – non significant). (**b**) CGNs were treated with 12.5 μM EGA or vehicle for 30 min at 37 °C. Then, neurons were incubated with BoNT/D (2.5 pM) at 4 °C for 15 min, washed and incubated at 37 °C with buffers at different pH value (7.4 or 4.5) for 10 min; after washing, the neurons were incubated for 24 h with standard medium in the presence of 50 nM bafilomycin A1 and where indicated EGA. Then the SNARE protein content was estimated by immunoblotting with specific antibodies. Values are reported as the ratio between the staining with the antibody specific for VAMP2 and the staining with an antibody specific for SNAP25, and normalized vs. non-treated neurons (NC). All data are presented as mean values and error bars indicated the standard deviation obtained from three independent experiments (ns – non significant).

Gillespie *et al.* (2013) reported that EGA prevents the toxicity of bacterial toxins and viruses by blocking their trafficking from early to late endosomes. Here, we have shown that EGA inhibits BoNTs without interfering with the main events along their mechanism of action. As a consequence, we reasoned that EGA could alter the trafficking of BoNTs after their internalization, possibly preventing them to reach their translocation-competent compartment. If it is the case, EGA should not be capable of inhibiting BoNTs toxicity when their trafficking is bypassed by inducing the entry of the L chain across the plasma membrane of neurons^36^. As this experimental approach strongly depends on the binding to both receptors at the plasma membrane^36^, we could perform this experiment only with BoNT/B, using an established PC12 cell line expressing on the plasma membrane the lumenal domain of Synaptotagmin I, the BoNT/B protein receptor^9^, and with BoNT/D, whose binding domain harbors two ganglioside binding sites^37^, using CGNs^36^. Figure 5a,b show that a low pH jump in the extracellular medium induces the translocation of BoNT/B and BoNT/D L chains across the plasma membrane as evaluated by the cleavage of VAMP2. In agreement with our hypothesis, the same experiment performed in the presence of EGA showed the identical activity of both BoNTs on VAMP2. This suggests that, when these neurotoxins bypass their canonical entry routes, EGA cannot impact on their activity anymore. Taken together the results presented here indicate that EGA prevents the activity of BoNTs by inhibiting their intraneuronal trafficking.

### EGA interferes with the neuroparalytic activity of BoNT/A, BoNT/B and BoNT/D at the mouse hemidiaphragm assay and *in vivo*

**Figure 6:**
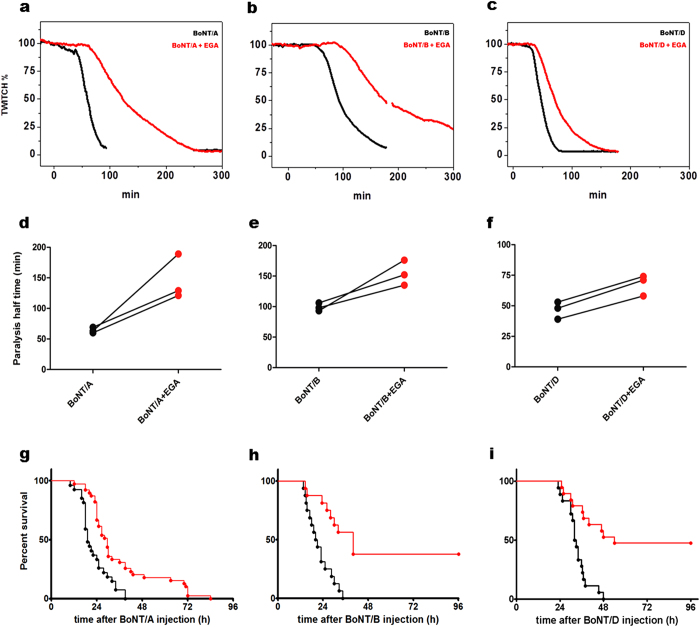
EGA provides protection against different serotypes of BoNTs in the phrenic nerve-hemidiaphragm twitch model, delays death induced by BoNT/A and strongly protect against death induced by BoNT/B and BoNT/D. (**a–c**) For each experiment, 12.5 μM EGA or vehicle (DMSO) was added to the nerve-muscle preparations in the bath at 37 °C; after 30 min, 10 pM BoNT/A (**a**) or 10 pM BoNT/B (**b**) or 100 pM BoNT/D (**c**) was added (time = 0). Muscle twitch was induced by nerve stimulation and monitored until paralysis. A representative experiment is reported for each toxin, showing the progressive twitch decrease in the presence of vehicle (black trace) or EGA (red trace). (**d–f**) Each experiment performed for (**a–c**) was expressed as the time required to decrease the twitch to 50% of the initial value (paralysis half time) in vehicle (black points) or EGA treated muscles (red points). (**g–i**) Adult CD1 mice preconditioned with EGA 12.5 mg/Kg (n = 20) or vehicle alone (n = 20) were i.p. injected with 2 × MLD_50_ of BoNT/A (**g**) or BoNT/B (**h**) or BoNT/D (**i**). The animals were monitored every 4 hrs for 96 hrs. The survival curves were compared and found to be significantly different (p < 0.0001).

The main aim of the present work was to test the inhibitory capacity of EGA against BoNTs toxicity *in vivo*. Therefore, after the *in vitro* approach, we used the mouse hemidiaphragm muscle paralysis model, an *ex vivo* preparation which represents the standard method to assay the neuroparalytic activity of BoNTs at the neuromuscular junction. In this experimental set up, BoNTs induce a decrease in the twitch capability of the diaphragmatic muscle by exerting its metalloprotease activity within the attached phrenic nerve. This decay is followed over time, and is used to evaluate BoNT potency, but can also be adapted to determine the inhibitory capacity of antitoxins^38^. As shown in Fig. 6(a–c, black traces), BoNT/A, BoNT/B and BoNT/D induce a rapid drop in the twitch capability of the diaphragm muscle. On the other hand, the pre-treatment with 12.5 μM EGA, strongly delays the neuroparalytic activity of the three BoNTs (red traces). This inhibitory effect can be appreciated also by comparing the different parameters reported in Table S1, and the t_50%_ values in particular (Fig. 6d–f), namely the time needed to halve the muscle twitch capacity, which results greatly increased by the treatment with the drug, and found to be significantly different (Table S1).

We then tested the inhibitory effect of EGA *in vivo*. A wide range of doses from 7.5 mg/kg to 40 mg/kg per day was administered via b.i.d. intraperitoneal injections in mice: even after one week of treatment with this regimen, the drug was well tolerated by mice which did not show any sign of decreased vitality in terms of breathing, eating and drinking nor in terms of motility as compared with vehicle injected controls. The lethality of our preparations of BoNT/A,/B and/D was evaluated in preliminary experiments, and a dose of 0.5 ng/kg (BoNT/A), 0.9 ng/kg (BoNT/B) and 0.045 ng/kg (BoNT/D) was sufficient to progressively induce the classical symptoms of botulism (fur ruffling, sides musculature collapse, generalized weakness, labored breathing) and cause the deadly respiratory failure within 48 hours post injection (black traces of Fig. 6 panels g–i). The red traces of the same figure (panels h and i) show that EGA is particularly efficacious in preventing death from botulism induced by BoNT/B and BoNT/D. Importantly, in those mice that eventually died, the symptoms occurred with delay and were less pronounced. This was the case also for BoNT/A injected mice, where symptoms developed later and were milder, but without a reduced toxin lethality (Fig. 6g, red trace).

## Discussion

The main result reported here is simple and very relevant at the same time. EGA is a potent inhibitor of the neuroparalytic activity of botulinum neurotoxins *in vitro* and *in vivo*, at doses that cause no apparent toxicity. This result indicates that EGA is the lead of a novel class of inhibitors potentially capable of preventing the activity of BoNTs in humans. This is the more relevant considering that the recent years have witnessed the discovery of a large number of novel BoNTs, with different immunoreactivity^2^,^39^,^40^, suggesting the possibility of the identification of BoNT variants that may be poorly neutralized by currently available antisera. This situation calls for the discovery of inhibitors capable of preventing the activity of all BoNTs. Necessarily, these novel inhibitors must be non-toxic to humans and must be effective *in vivo*. Notwithstanding long efforts of many laboratories, this goal has only partially been achieved^41^. We recently reported on inhibitors of the Thioredoxin reductase–Thioredoxin redox couple that effectively prevent the neuroparalytic activity of all BoNT serotypes without causing toxic effects in mice^19^,^20^,^42^

Here we add another lead compound with a different mechanism of inhibition. Despite our efforts using primary cultures of neurons and neuromuscular junction preparation, we have not identified the target of EGA, but we did not note toxic effects in mice treated with a dose that largely prevents the action of the three BoNTs used here. We have found that the main steps of BoNTs mechanism of action, i.e. binding, internalization, acidification of intracellular compartment, L chain translocation, disulphide reduction and substrate proteolysis, are not affected by this compound (Figs 4 and 5, Figure S2 and Figure S3). Notably, the range of concentration that block BoNTs in cultured neurons is the same previously found to inhibit the toxicity of different toxins and viruses in primary and immortalized macrophages. This suggests that, rather than having a direct effect on BoNTs (or on the other pathogens), EGA interferes with an intracellular host target responsible for their trafficking. This conclusion is reinforced by the result showing that EGA had no effect on the translocation of the L chain from the plasma membrane, when the canonical internalization route was bypassed (Fig. 5).

All known protein receptors of BoNTs are the lumenal domains of integral proteins of synaptic vesicles which suggests the general conclusion that all BoNTs are endocytosed inside these organelles at nerve terminals. However, the following trafficking of synaptic vesicles is not fully understood, though there is evidence that they may fuse with synaptic endosomes where they are quality controlled and then released to re-enter the synaptic vesicle cycle^43^,^44^,^45^,^46^,^47^,^48^. As a consequence, the fact that the three different serotypes considered here are differently protected by EGA, which inhibits the maturation of early endosomes^27^, is an interesting aspect of the current study, because it revives the possibility that different BoNT may be trafficked through different routes inside the nerve terminals. Indeed, the diverse protein receptors of BoNTs may account for distinct fates of each toxin-receptor complexes, which have not yet been determined case by case. An alternative explanation is suggested by the finding that part of BoNT/A may enter terminals independently from SVs endocytosis^31^,^32^, which is supported by studies showing that BoNTs display toxicity independently of the stimulation of SVs recycling^34^,^49^,^50^,^51^,^52^,^53^. The fact that EGA completely inhibits the activity of BoNT/B, although used at a concentration much higher than that of BoNT/A, opens the possibility that the activity of this toxin is dependent on a trafficking through endosomes and does not translocate its catalytic part into the cytosol across the SV membrane. This is a surprising finding which was unexpected on the basis of the knowledge that the SV protein synaptotagmin mediates the entry of BoNT/B^8^,^9^. However, considering that synaptotagmin can be trafficked through early endosome^54^, the possibility that also BoNT/B may need the passage through this organelle to reach a membrane translocation-competent compartment becomes plausible. It is also in keeping with its slow time course of entry into cultured neurons as compared with other serotypes^34^,^55^. Moreover, a considerable amount of synaptotagmin molecules remains exposed on the plasma membrane surface, in a steady-state with those recycled through sorting endosomes^56^, which makes possible that BoNT/B forms a toxin-receptor complex on the plasma membrane, rather than within SVs. This fits well with the present findings that: i) the internalization of c-Myc-HC/B was much less affected compared to that of cpV-HC/A, by the pre-treatment with BoNT/D (Fig. 4b and Figure S2b) and ii) the different staining pattern of the BoNT/A and BoNT/B binding domains (Fig. 4a and Figure S2a). This possibility is also supported by the *in vivo* finding that EGA has a remarkable effect against the lethality of BoNT/B and a lower one on BoNT/A (Fig. 6g,h).

The behavior of BoNT/D in response to inhibition of the endosomal pathway by EGA, in cultured neurons is more similar to that of BoNT/A rather than BoNT/B, as VAMP2 cleavage was not completely prevented (Figs 2c and 3e,f). On the other hand, BoNT/D was efficaciously inhibited by EGA *in vivo,* with an inhibitory profile similar to that of BoNT/B (Fig. 6i). The mechanism of BoNT/D binding to neurons is poorly understood and therefore its internalization and trafficking properties are not entirely clear^37^,^57^, and as a consequence it is even more difficult to envisage how this toxin could be internalized and trafficked. The obtained results clearly show that the observations of cell culture experiments cannot be transferred *tout court* to *in vivo* conditions.

The present lack of knowledge on the biochemical target of EGA does not prevent research aimed at finding more potent inhibitors of the BoNT neuroparalytic action. Clearly, EGA action is a preventive one, as it cannot affect those L chains that have already translocated in the cytosol. Nevertheless, it can alleviate the symptoms of botulism after diagnosis because a considerable amount of BoNT remains in the general circulation of botulism patient for weeks after the first diagnosis^58^,^59^,^60^. Perhaps, more importantly, the present findings are relevant for infant botulism where a continuous entry of BoNT into the general circulation occurs via adsorption of the toxin produced by Clostridia that have colonized the gastrointestinal tract of infants owing to the reduced intestinal flora competing with Clostridia^2^,^61^.

We would like to conclude by pointing out that the search for novel EGA-derived analogues is made simpler by the design of the novel method of synthesis of this compound described here, which provides a much higher yield with respect to the recently described method^29^. This procedure allowed us to rapidly and efficiently synthesize large quantities of EGA, an essential pre-requisite to produce the considerable amount necessary for a possible employment of this or related compounds in humans.

## Methods

### Chemical Synthesis

Detailed protocol for EGA chemical synthesis is available in Supplementary Information.

### Botulinum neurotoxin inhibition assay

EGA was dissolved in DMSO to prepare a stock solution (12.5 mM). CGNs at 6–8 days *in vitro* (DIV) were treated for 30 min with the indicated concentrations of EGA in complete culture medium at 37 °C and 5% CO_2_. 0.3 nM BoNT/A, 5 nM BoNT/B or 0.025 pM BoNT/D was added, in the presence of the same concentration of inhibitor, and left for 12 hr at 37 °C and 5% CO_2_. Further details can be found in the Supplementary Information.

### cpV-HC/A and c-Myc-HC/B binding assay

CGNs were treated with EGA 12.5 μM or vehicle (DMSO) in culture medium at 37 °C. After 30 min, for immunocytochemistry experiments, 100 nM cpV-HC/A or c-Myc-HC/B was added in stimulating culture medium (complete culture medium, 57 mM KCl), for 1 hr. The same protocol was used with 250 nM of cpV-HC/A or c-Myc-HC/B but neurons were then lysed and immunoblotted to obtain a quantitative result. Details are in the Supplementary Information.

### Low pH induced translocation of BoNT/B and BoNT/D across the plasma membrane

Experiment was conducted as previously described^36^. Detailed protocol is available in Supplementary Information.

### Mouse diaphragm and lethality assay

All experiments were performed in accordance with the European Communities Council Directive n° 2010/63/UE and approved by the Italian Ministry of Health. Mouse diaphragms were isolated from CD-1 mice weighing about 20–25 g and halved into two contralateral hemi-diaphragms still innervated with the own phrenic nerve, and were treated as described in the Supplemental Experimental Procedures. Lethality assays were performed using Swiss-Webster adult male CD1 mice weighing 26–28 g as described in Supplementary Information.

### Statistical analysis

For all the experiments, data are presented as mean values. Bars indicated the standard deviation. Significance was calculated by Student’s t test (unpaired, two-side). *p < 0.05, **p < 0.01, ***p < 0.0001. Only values below 0.05 were considered significant (ns – non significant).

## Additional Information

**How to cite this article**: Azarnia Tehran, D. *et al.* A Novel Inhibitor Prevents the Peripheral Neuroparalysis of Botulinum Neurotoxins. *Sci. Rep.* **5**, 17513; doi: 10.1038/srep17513 (2015).

## Supplementary Material

Supplementary Information
