# Supplementary material for: A Novel Inhibitor Prevents the Peripheral Neuroparalysis of Botulinum Neurotoxins
Source: Sci Rep. 2015 Dec 16;5:17513. doi: 10.1038/srep17513 (PMC4680858; doi:10.1038/srep17513)
Supplement: Supplementary Information [file srep17513-s1.pdf]

A Novel Inhibitor Prevents the Peripheral Neuroparalysis of Botulinum Neurotoxins

Domenico AZARNIA TEHRAN[1,†], Giulia ZANETTI[1,†], Oneda LEKA[1], Florigio LISTA[3], Silvia FILLO[3], Thomas BINZ[4], Clifford C. SHONE[5], Ornella ROSSETTO[1], Cesare MONTECUCCO[1,2], Cristina PARADISI[6], Andrea MATTAREI[6,*] and Marco PIRAZZINI[1,*]

Supplementary figures

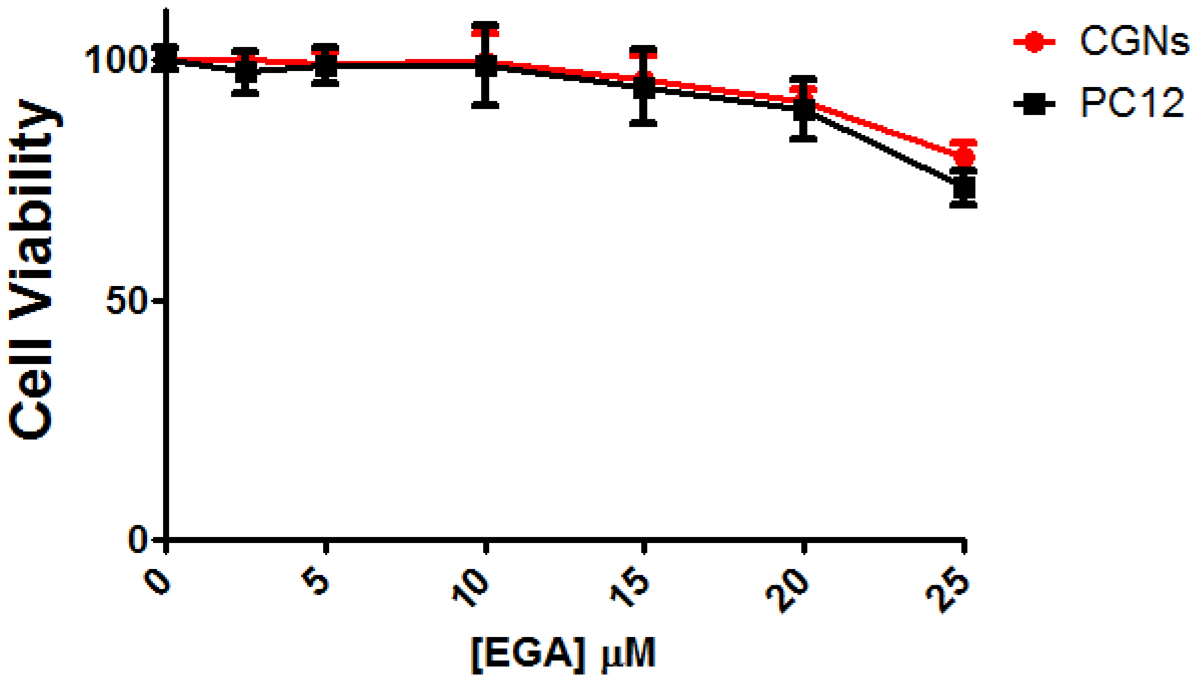

Figure S1. EGA does not affect cell viability of CGNs and PC12. CGNs and PC12 were treated with increasing concentration of EGA ranging from 2.5 to 25 μM or vehicle in culture medium at 37° C. After 24 hours, cell viability has been assayed with a MTS assay. Data are presented as a percentage with respect to cells treated with the vehicle, set as 100%. All data are presented as mean values and error bars indicated the deviation standard obtained from three independent experiments.

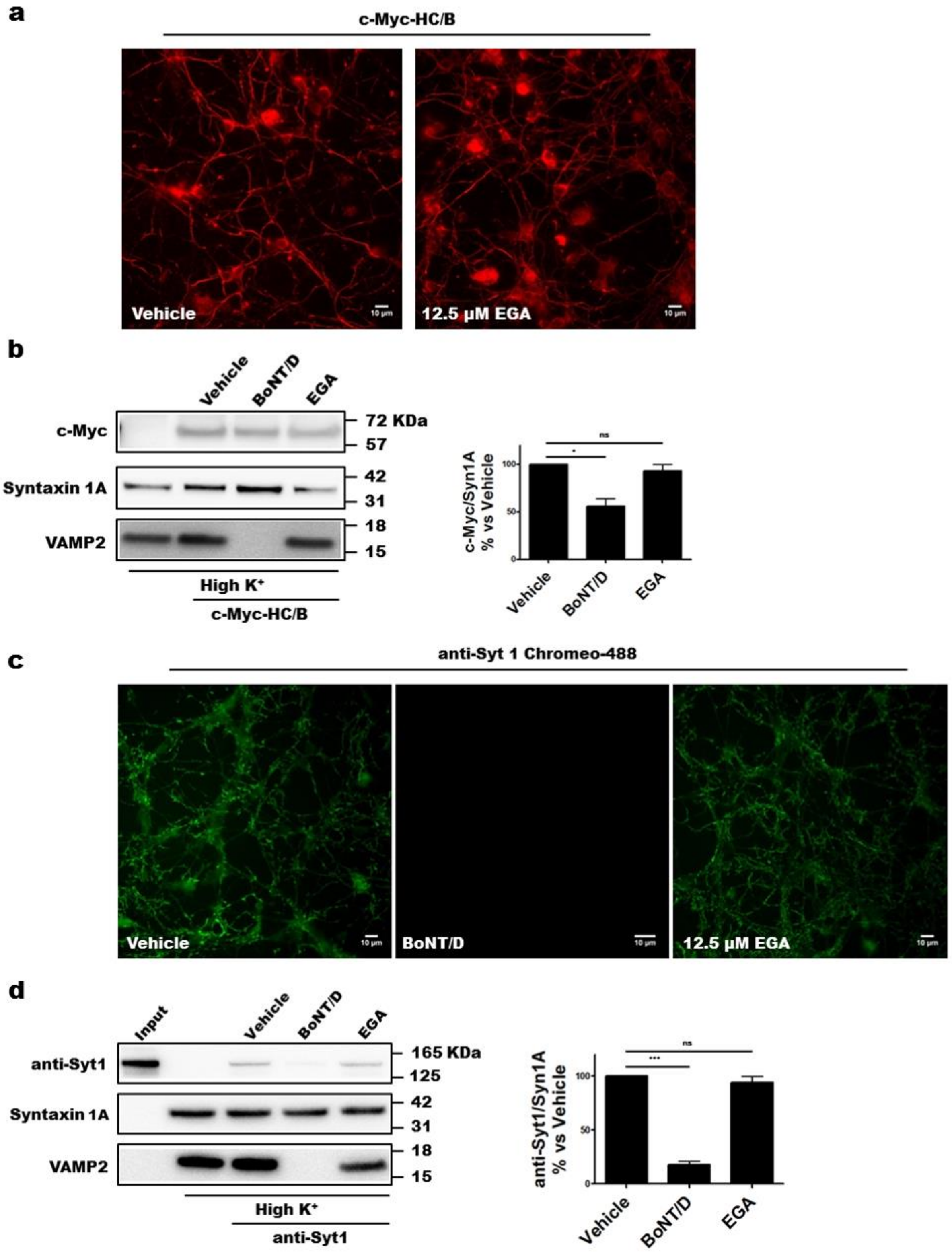
a
c-Myc-HC/B
Vehicle
12.5 µM EGA
10 µm
b
Vehicle
BoNT/D
EGA
c-Myc
Syntaxin 1A
VAMP2
72 KDa
57
42
31
18
15
High K⁺
c-Myc-HC/B
c-Myc/Syn1A % vs Vehicle
ns
*
100
50
0
c
anti-Syt 1 Chromeo-488
Vehicle
BoNT/D
12.5 µM EGA
10 µm
d
Input
Vehicle
BoNT/D
EGA
anti-Syt1
Syntaxin 1A
VAMP2
165 KDa
125
42
31
18
15
High K⁺
anti-Syt1
anti-Syt1/Syn1A % vs Vehicle
ns
***
100
50
0

Figure S2. EGA does not affect binding and synaptic vesicles dynamics in CGNs. (a) CGNs were treated with EGA (12.5 µM) or vehicle in culture medium at 37° C. After 30 min, 100 nM c-Myc-HC/B was added in high K^+ buffer for 1 h. Neurons were then washed, fixed, permeabilized and stained with a primary antibody specific for the c-Myc epitope. An Alexa Fluor 488 goat anti-mouse secondary antibody was used for detection. These images are representative of two independent sets of experiments. Scale bar, 10 µm. (b) CGNs were treated as in *(a)* with 250 nM of c-Myc-HC/B and then lysed. The c-Myc-HC/B content was estimated with specific antibodies against c-Myc epitope. Syntaxin 1A was used as loading control and VAMP2 to assess BoNT/D cleavage. The amount of c-Myc-HC/B was determined as a ratio to Syntaxin 1A staining taking the value in non-treated cells (vehicle) as 100%. All data are presented as mean values and error bars indicated the standard deviation obtained from two independent experiments (* $p<0.05$; ns – non significant). (c) CGNs were treated with EGA (12.5 µM) or vehicle at 37° C for 30 minutes. Where indicated, neurons were pre-treated with BoNT/D (10 nM) for 30 min. Cells were then incubated for 20 min with an antibody against the lumenal domain of Synaptotagmin-1 conjugated to Chromeo 488 in high K^+ buffer. At the end of the incubation, CGNs were washed twice, fixed and imaged by fluorescence microscopy. These images are representative of three independent sets of experiments. Scale bar, 10 µm. (d) CGNs were treated as in (c), using a non-fluorescent version of the same anti-Synaptotagmin-1 antibody. At the end of the incubation, neurons were washed twice and lysed in non-reducing Laemmli sample buffer. In the upper panel the internalized antibody was detected by immunoblotting, using an anti mouse HRP-conjugated secondary antibody. Blots were then stripped and incubated with specific antibodies against VAMP2 to assess BoNT/D cleavage and against Syntaxin 1A as loading control. In the first lane (input) 50 ng of the anti-Synaptotagmin 1 antibody were loaded as reference. The bottom panel reports the quantification. All data are presented as mean values and error bars indicated the

standard deviation obtained from three independent experiments (*** p<0.0001; ns – non significant).

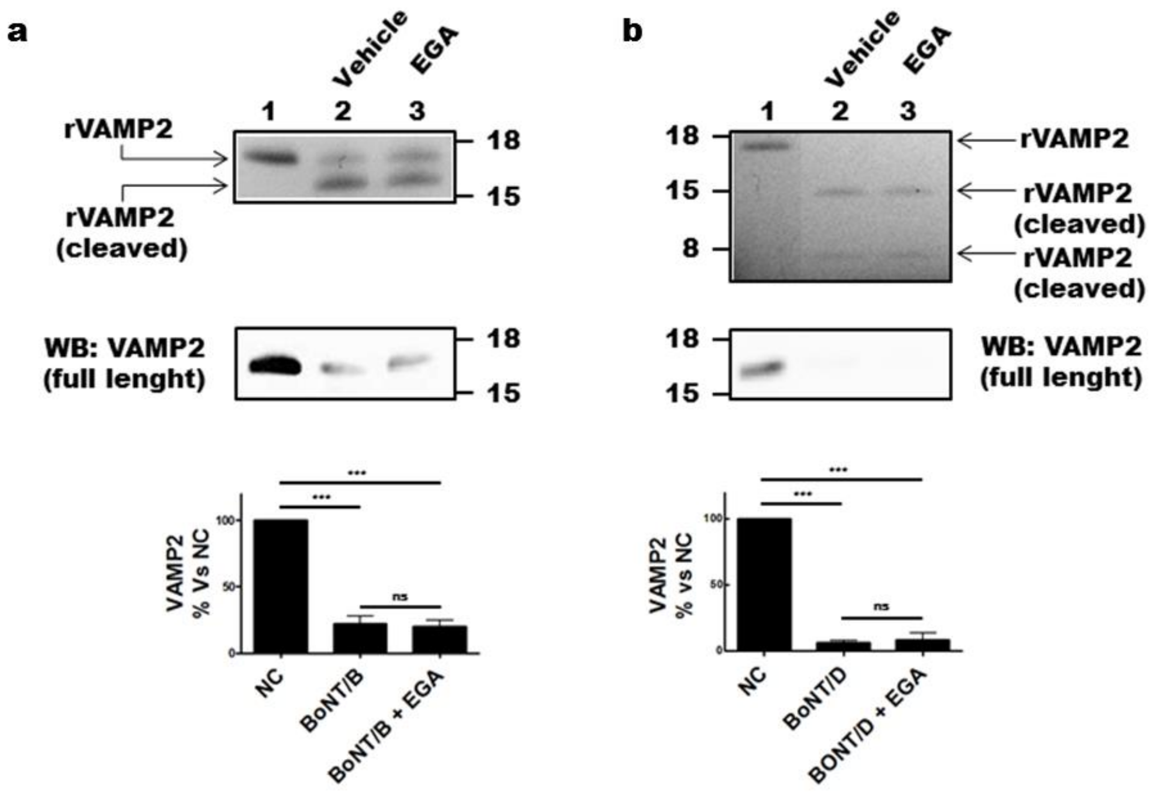

Figure S3. EGA does not affect BoNT/B and /D metalloprotease activity in vitro. (a) BoNT/B (1 µg) or (b) BoNT/D (0.25 µg) was reduced in the presence of EGA (12.5 µM) for 30 min at 37 °C. 1 µg of recombinant VAMP2 (rVAMP2) was then added, the concentration of inhibitor restored and the reaction carried out for 12 hours at 37 °C. VAMP2 cleavage was assessed by SDS-PAGE (top panels) or immunoblotting (middle panels) with an antibody that recognizes full length VAMP2. Lower panels show the densitometry analysis of western blots. All data are presented as mean values and error bars indicated the standard deviation obtained from three independent experiments (*** p<0.0001; ns – non significant).

	BoNT/A		BoNT/B		BoNT/D	
	**DMSO**	**EGA**	**DMSO**	**EGA**	**DMSO**	**EGA**
**Lag phase (minutes)**	36.3 ± 3.37	59.6 ± 0.9**	56.3 ± 2.4	82.0 ± 5.3*	29.0 ± 5.1	34.7 ± 2.4^{ns}
**$t_{1/2}$ (minutes)**	64.0 ± 2.6	146.3 ± 21.5*	99.0 ± 3.8	154.3 ± 11.9*	46.7 ± 4.1	67.7 ± 4.9*
**Slope**	2.1 ± 0.14	0.5 ± 0.09**	1.3 ± 0.11	0.6 ± 0.01**	2.4 ± 0.16	1.46 ± 0.09**
**N**	3	3	3	3	3	3

Table S1. Statistical analysis of the phrenic nerve-hemidiaphram twitch model. Given the variability of each muscle contraction, the data from BoNT/A, /B and /D intoxicated muscles treated with EGA or DMSO, were normalized and each single group was compared. The average values±SEM of lag phase, $t_{1/2}$ and slope derive from three independent experiments. Significance was calculated by Student's t test (unpaired, two-side). *P < 0.05; **P < 0.01. Only values below 0.05 were considered significant.

Supplementary experimental procedures

Reagents. BoNT/A was purified as previously described[1,2]. BoNT/B and BoNT/D were produced in *E. coli* as recombinant proteins[3,4]. LysoTracker® Red DND-99 was purchased from ThermoFischer Scientific (L-7528), instead, Bafilomycin A1 (sc-201550) from Santa Cruz Biotechnology. Cytosine β-D-arabinofuranoside hydrochloride (C6645), DNAse I from bovine pancreas (DN25), poly-L-lysine hydrobromide (P1274), solvents and reagents were purchased from Sigma Aldrich, and were used as received.

Chemical Synthesis. Phenyl (2,6-dimethylphenyl)carbamate (**2**): 2,6-dimethylaniline (1.00 g, 8.3 mmol, 1.0 eq) was dissolved in DCM (15 mL) and pyridine (0.73 g, 9.2 mmol, 1.1 eq) and cooled on ice-bath. Phenyl chloroformate (1.43 g, 9.2 mmol, 1.1 eq) in DCM (25 mL) was added dropwise. After addition the flask was brought to room temperature and stirred for 3 h. The reaction mixture was diluted with DCM (50 mL) and washed with 0.5 N HCl (5 × 100 mL). The organic layer was dried over MgSO4 and concentrated. The resulting crude product was purified by flash column chromatography on silica gel (eluent: Petroleum Ether/DCM/Acetone 60:35:5) to afford **2** as a bright white solid (1.92 g, 8.0 mmol, 96%). ^{1}H NMR (500 MHz, DMSO) δ 9.35 (s, 1H, -N*H*-), 7.43 (m, 2H, 2 × Ar-*H*), 7.24 (s, 1H, 1 × Ar-*H*), 7.22 (m, 2H, 2× Ar-*H*), 7.21 (m, 2H, 2 × Ar-*H*), 7.12 (m, 3H, 3 × Ar-*H*), 2.28 (s, 6H, 2 × -C*H3*); ^{13}C NMR (126 MHz, DMSO) δ 152.95, 151.56, 136.01, 134.76, 129.86, 128.38, 127.23, 125.56, 122.20, 18.47; ESI-MS: m/z = 242 = $[M+H]^+$; HRMS (ESI^+): m/z 242.1193 $[M+H]^+$, calculated for $C_{15}H_{16}NO_2$: 242.1181. 2-(4-Bromobenzylidene)-N-(2,6-dimethylphenyl)hydrazinecarboxamide (3, *EGA*): 2 (0.50 g, 2.1 mmol, 1.0 eq) was dissolved in DME (5 mL) and mixed with hydrazine monohydrate (50-60%, 0.13 g, 4.2 mmol, 2.0 eq) at 0°C. After addition the flask was brought to rt and stirred for 24 h. The reaction mixture was concentrated to give a crude white solid that was used

without further purification. This white solid was dissolved in chloroform (20 mL), mixed with 4-bromobenzaldehyde (0.77 g, 4.2 mmol, 2.0 eq) and vigorously stirred 15 h at room temperature. Solvent was removed under reduced pressure and the crude was purified by flash column chromatography on silica gel (eluent: DCM/EtOAc 85:15) to afford EGA (3) as a bright white solid (0.63 g, 1.8 mmol, 88%). ^{1}H NMR (500 MHz, DMSO) δ 10.65 (s, 1H, -N*H*-), 8.58 (s, 1H, -C*H*=N-), 7.88 (s, 1H, -N*H*-), 7.82-7.80 (d, 2H, 2 × Ar-*H*), 7.59-7.57 (d, 2H, 2 × Ar-*H*), 7.09 (m, 3H, 3 × Ar-*H*), 2.20 (s, 6H, 2 × -CH_3). ^{13}C NMR (126 MHz, DMSO) δ 154.20, 138.99, 136.71, 136.02, 134.59, 131.94, 129.24, 128.01, 126.67, 122.70, 18.68. ESI-MS: m/z = 346 = $[M+H]^+$; HRMS (ESI^+): m/z 346.0563 $[M+H]^+$, calcd for $C_{16}H_{17}BrN_3O$: 346.0555. TLCs were run on silica gel supported on plastic (Macherey-Nagel Polygram®SIL G/UV_{254}, silica thickness 0.2 mm) and visualized by UV detection. Flash chromatography was performed on silica gel (Macherey-Nagel 60, 230-400 mesh granulometry (0.063-0.040 mm)) under air pressure. The solvents were analytical or synthetic grade and were used without further purification. ^{1}H NMR spectra were recorded with a Bruker AVII500 spectrometer operating at 500 MHz. Chemical shifts (δ) are given in ppm relative to the signal of the solvent. Mass spectra were performed with a 1100 Series Agilent Technologies system, equipped with binary pump (G1312A) and MSD SL Trap mass spectrometer (G2445D SL) with ESI source. ESI-MS positive spectra of reaction intermediates and the final purified product were obtained from solutions in acetonitrile, eluting with a water:acetonitrile, 1:1 mixture containing 0.1% formic acid. High-resolution mass measurements were obtained using a Mariner ESI-TOF spectrometer (PerSeptive Biosystems). HPLC-MS analysis was used to confirm the purity (> 95%).

Cerebellar Granule Neurons (CGN) cultures. Primary cultures of rat cerebellar granule neurons (CGNs) were prepared from 6- to 8-days-old rats[5]. Cerebella were isolated, mechanically disrupted and then trypsinized in the presence of DNase I. Cells were then

plated into 24 well plates, pre-coated with poly-L-lysine (50 µg/mL), at a cell density of 4 x 10^5 cells per well. Cultures were maintained at 37 °C, 5% CO_2, 95% humidity in BME supplemented with 10% fetal bovine serum, 25 mM KCl, 2 mM glutamine and 50 µg/mL gentamicin (hereafter indicated as complete culture medium). To arrest growth of non-neuronal cells, cytosine arabinoside (10 µM) was added to the medium 18–24 h after plating.

Botulinum neurotoxin inhibition assay and immunocytochemistry. CGNs at 6–8 DIV were treated for 30 min with 12.5 µM of EGA or vehicle (DMSO) in complete culture medium at 37 °C and 5% CO_2. 0.3 nM BoNT/A or 5 nM BoNT/B or 0.025 pM BoNT/D was added and left for 12 hours at 37 °C and 5% CO_2. Neurons were then washed with PBS, fixed for 10 minutes at RT with 4% paraformaldehyde in PBS, quenched (50 mM NH_4Cl in PBS) for 20 minutes and permeabilized with 5% acetic acid in ethanol for 20 minutes at -20° C. CGNs were then incubated with indicated primary antibodies. BoNT/A cleavage was evaluated following the generation of the cleaved form of SNAP25, whereas the cleavage of BoNT/B and BoNT/D was evaluated following the disappearance of the staining due to a primary antibody recognizing the full-length form of VAMP2 (Synaptic System, 104 211). As internal control (not shown) it was used an anti SV2A (Santa Cruz, [E-8] sc376234) or anti SV2B (Synaptic System, 119 102), respectively. Primary antibodies were detected with Alexa Fluor 488 goat anti-mouse IgG (Life Technologies, A-11001) and Alexa Fluor 555 goat anti-rabbit IgG (Life Technologies, A-21428). Coverslips were mounted using Fluorescent Mounting Medium (Dako) and examined by epifluorescence (Leica CTR6000) microscopy. Images were collected with the same lamp intensity and exposure time.

For immunoblotting experiments, the neurons were treated with the same condition and the neurotoxicity was evaluated following the specific proteolytic activity of the toxin with

specific antibodies against their SNARE protein targets: anti BoNT/A-cleaved SNAP25[6], anti SNAP25 (SMI81: abcam, ab24737), anti VAMP2 (Synaptic System, 104 211) and anti BoNT/B-cleaved form of VAMP2[7]. The staining with anti Na^+/K^+ ATPase (abcam, ab7671) was used as loading control.

Immunoblotting. Cells were directly lysed with reducing Laemmli sample buffer containing protease inhibitors (complete Mini EDTA-free, Roche). Protein concentration was determined with the BCA test (Pierce BCA protein assay, Thermo Scientific), and equal amounts were loaded onto a 4-12% NuPage gel or 12% NuPage gel (Life technologies) and separated by electrophoresis in 1X MES buffer or 1X MOPS (Life technologies), respectively. Proteins were then transferred onto Protran nitrocellulose membranes (Whatman) and saturated for 1 h in PBST (PBS 0.1% Tween20) supplemented with 5% non-fatty milk. Incubation with primary antibodies was performed overnight at 4°C. The membranes were then washed three times with PBST and incubated with secondary HRP-conjugated antibodies (goat anti-mouse IgG, H&L chain specific peroxidase conjugate, Merk Millipore 401215 and goat anti-rabbit IgG, H&L chain specific peroxidase conjugate, Merk Millipore 401393). Finally, membranes were washed twice with PBST and once with PBS; visualization was carried out using Luminata Crescendo (Merck Millipore).

cpV-HC/A and c-Myc-HC/B expression, purification and binding assay. The HC of BoNT/B (nucleotides corresponding to residues 833-1291) with a N-terminus c-Myc tag was cloned into a pRSETa His-tag vector (Novagen) and expressed into BL21pLysS *E.coli* cells. Protein purification was achieved by affinity chromatography with a prepacked HisTrap Ni column (GE Healthcare) and then by size-exclusion chromatography using a Superdex 200, 10/300GL column (GE Healthcare). The purified c-Myc-HC/B fusion protein

were pooled and concentrated using a membrane filter with a cutoff of 30 kDa (Amicon Millipore).

The HC of BoNT/A (nucleotides corresponding residues 876-1296) fused with cpV (Circularly Permuted Venus) at the N-terminus was cloned into a pET28a His-tag vector (Novagen) and expressed in BL21 DE3 *E.coli* cells. Purification of cpV-HC/A fusion protein was achieved as described for c-Myc-HC/B.

In binding and internalization assay, CGNs were treated with EGA 12.5 µM or vehicle (DMSO) in culture medium at 37° C. After 30 minutes, 100 nM cpV-HC/A or c-Myc-HC/B was added in stimulating culture medium (complete culture medium, 57 mM KCl), for 1 hr. Neurons were then washed twice, fixed with 4% paraformaldehyde in PBS and directly imaged for cpV or permeabilized and stained with a primary antibody specific for the c-Myc epitope (Sigma Aldrich, M4439). An Alexa Fluor 488 goat anti-mouse (Life Technologies, A-11001) was used to detect the c-Myc primary antibody.

The same experiment was performed with 250 nM of cpV-HC/A or c-Myc-HC/B but neurons were then lysed and immunoblotted to obtain a quantitative result. Where indicated, neurons were pre-treated with 10 nM of BoNT/D for 30 minutes. Syntaxin 1A (Synaptic System, 110 111) staining was used as loading control, instead, VAMP2 (Synaptic System, 104 211) staining to assess BoNT/D cleavage. cpV-HC/A was detected with an anti-GFP antibody (Cell Signaling, #2956) whereas c-Myc-HC/B was detected with the aforementioned antibody.

Maturation of acidic compartment assay. CGNs at 6–8 DIV were treated for 30 min with the indicated concentrations of EGA or 10 nM Bafilomycin A1 in complete culture medium supplemented with 6.25 mM Hepes at 37 °C and 5% CO_2. 75 nM Lysotracker® Red DND-99 was added for 90 minutes. Cells were then washed with Krebs-Ringer buffer (KRH: 128 mM NaCl, 2.5 mM HEPES, 4.8 mM KCl, 1.3 mM $CaCl_2$, 1.2 mM $MgSO_4$ and 1.2 mM

K_2HPO_4) and images of living neurons were acquired with a Leica CTR6000 microscope. Fluorescence intensity was quantified using ImageJ software.

In vitro proteolytic activity. 0.25 µg BoNT/A or BoNT/D or 1 µg BoNT/B was incubated in reducing buffer (150 mM NaCl, 10 mM NaH2PO4, 15 mM DTT pH 7.4) in the presence of 12.5 µM of EGA for 30 min at 37 °C or DMSO. Then 1 µg of recombinant GST-SNAP25 or recombinant VAMP2 (1-96) was added to the reduced toxins, the concentration of inhibitor was restored, and the reaction was carried out for 12 hours at 37 °C. SNAP25 or VAMP2 cleavage was assessed by SDS-PAGE or immunoblotting using an anti-SNAP25 A-cleaved form or an anti-VAMP2 (Synaptic System, 104 211).

Low pH induced translocation of BoNT/B and BoNT/D across the plasma membrane. At a glance, 10^4 PC12-SYT N24Q were plated into 12-wells plates and maintained in RPMI supplemented with 10% HS, 5% FBS, 2 mM L-alanyl-L-glutamine (GlutaMAX), 100 U/ml penicillin, 100 µg/ml streptomycin, 250 ng/ml amphotericin B at 37 °C in a humid incubator. After adhesion, cells were incubated with a mixture of ganglioside (50 ug) for 24 hours. Cells were washed twice with culture medium and subsequently incubated with 10 nM of BoNT/B in ice-cooled medium (pH 7.4) and left at 4 °C for 15 minutes. After washing twice with the same cold medium, pre-warmed (37 °C) medium A (123 mM NaCl, 6 mM KCl, 0.8 mM $MgCl_2$, 1.5 mM $CaCl_2$, 5 mM NaP_i, 5 mM citric acid, 5.6 mM glucose, 10 mM NH_4Cl) - adjusted at indicated pH (7.4 or 4.5) with 1 M TRIS-base - was added and left for 10 minutes. Cells where then washed twice and further incubated in normal culture medium (pH 7.4) containing 50 nM Bafilomycin A1 for 24 hours. Where indicated, 12.5 µM EGA was pre-incubated with cells for 30 minutes and was then present in all solutions used along experiment. The same procedure was performed using CGNs, 2.5 pM of BoNT/D and after low pH assay the incubation was prolonged for 24 hr. Where indicated, 12.5 µM EGA was pre-incubated with cells for 30 minutes and was then present in all

solutions used along experiment. The translocation of BoNT/B or BoNT/D was assessed by following the metalloprotease activity against VAMP2 via western blot using an anti-VAMP2 (Synaptic System, 104211). The staining of SNAP25 (SMI81: abcam, ab24737), was used as loading control.

Mouse diaphragm and lethality assay. Muscles were mounted into two chambers filled with 4 ml of oxygenated (95% O_2, 5% CO_2) solution (139 mM NaCl, 12 mM $NaHCO_3$, 4 mM KCl, 2 mM $CaCl_2$, 1 mM $MgCl_2$, 1 mM KH_2PO_4 and 11 mM glucose, pH 7.4). The phrenic nerves were stimulated via two ring platinum electrodes with supramaximal stimuli of 3 V amplitude and 0.1 ms pulse duration, with a frequency of 0.1 Hz (Stimulator 6002, Harvard Apparatus, Massachusetts, USA). Muscle contraction was monitored with an isometric transducer (Harvard Apparatus); data was recorded and analysed via an i-WORX 118 system with Labscribe software (Harvard Apparatus). EGA was added directly to the oxygenated solution of one muscle to reach the final concentration of 12.5 µM of and the same volume of vehicle (DMSO) was added to the contralateral one for direct comparison. After 30 minutes of incubation, 10 pM BoNT/A or BoNT/B or 100 pM BoNT/D was added to both preparations and the twitch monitored until complete paralysis was achieved. Graphs show muscle twitching capability over time, reported as percentage with respect to the initial value obtained before toxin addition.

For lethality assay, EGA was dissolved in DMSO as a stock solution (12.5 mg/ml). Mice were conditioned for 3 days, with 12.5 mg/kg EGA or vehicle with intraperitoneal (i.p.) injections b.i.d. (every 12 hours). After the last injection of drug (or vehicle), mice were weighted and i.p. injected with 1 µl/g body weight of BoNT/A, /B or /D prepared as stock solutions (BoNT/A 0.5 pg/µl, BoNT/B 0.9 pg/µl and BoNT/D 0.045 pg/µl in 0.9% NaCl with 0.2% gelatin) roughly corresponding to $2XMLD_{50}$. Mice were monitored every 4 hr for 96

hr, after which the experiment was considered ended. Results are displayed as Kaplan-Meyer plots, and analysed with a Mantel-Cox test for statistical significance.

Synaptic vesicles dynamics assay. Experiment was performed as previously described [8]. Briefly, CGNs were conditioned in high K^+ buffer (70 mM NaCl, 2.5 mM HEPES, 57 mM KCl, 1.3 mM $CaCl_2$, 1.2 mM $MgSO_4$ and 1.2 mM K_2HPO_4) for 10 minutes. Hereafter, 5 µg/ml of Chromeo 488 (Synaptic System, 105 311CR1) was added for 20 min in the same buffer. Cells were then washed twice with PBS and fixed. Internalized antibodies were imaged using a Leica CTR6000 microscope. Where indicated, 10 nM BoNT/D or 12.5 µM of EGA (or DMSO) were pre-incubated in normal culture medium for 30 minutes. The same concentration of EGA is maintained during antibody incubation. In order to have a quantitative result, the same experiment was performed using as a read-out western blot. Accordingly, CGNs were lysed in non-reducing condition, blotted on nitrocellulose membrane saturated for 1 hr in PBST supplemented with 5% non-fatty milk and directly incubated with secondary antibody to detect the internalized anti-Synaptotagmin1 antibody (Synaptic System, 105 101). The staining of VAMP2 (Synaptic System, 104 211) was used to assess BoNT/D cleavage, instead, Syntaxin 1A (Synaptic System, 110 111) was used as loading control.

Viability test. CGNs or PC12-SYT N24Q were seeded in a 96 wells plates at a cell density 10^5 cells per well. Different concentration of EGA, ranging from 0 to 25 µM, were added and left for 24 hours. Neurons were then washed and MTS assay (Promega) performed according to manufacturer indication.

References

1 Schiavo, G. & Montecucco, C. Tetanus and botulism neurotoxins: isolation and assay. *Methods Enzymol* **248**, 643-652. (1995).

2 Shone, C. C. & Tranter, H. S. Growth of clostridia and preparation of their neurotoxins. *Curr Top Microbiol Immunol* **195**, 143-160. (1995).

3 Rummel, A., Mahrhold, S., Bigalke, H. & Binz, T. The HCC-domain of botulinum neurotoxins A and B exhibits a singular ganglioside binding site displaying serotype specific carbohydrate interaction. *Mol Microbiol.* **51**, 631-643. (2004).

4 Bade, S. *et al.* Botulinum neurotoxin type D enables cytosolic delivery of enzymatically active cargo proteins to neurones via unfolded translocation intermediates. *J Neurochem* **91**, 1461-1472. (2004).

5 Rigoni, M. *et al.* Snake presynaptic neurotoxins with phospholipase A2 activity induce punctate swellings of neurites and exocytosis of synaptic vesicles. *J Cell Sci.* **117**, 3561-3570. (2004).

6 Antonucci, F., Rossi, C., Gianfranceschi, L., Rossetto, O. & Caleo, M. Long-distance retrograde effects of botulinum neurotoxin A. *J Neurosci.* **28**, 3689-3696. (2008).

7 Mainardi, M., Pietrasanta, M., Vannini, E., Rossetto, O. & Caleo, M. Tetanus neurotoxin-induced epilepsy in mouse visual cortex. *Epilepsia* **53**, e132-136. (2012).

8 Kraszewski, K. *et al.* Synaptic vesicle dynamics in living cultured hippocampal neurons visualized with CY3-conjugated antibodies directed against the lumenal domain of synaptotagmin. *The Journal of Neuroscience* **15**, 4328-4342 (1995).
